# Current Understanding of the Structure and Function of Fungal Immunomodulatory Proteins

**DOI:** 10.3389/fnut.2020.00132

**Published:** 2020-08-18

**Authors:** Yusi Liu, Shanna Bastiaan-Net, Harry J. Wichers

**Affiliations:** ^1^Laboratory of Food Enzyme Engineering, Institute of Food Science and Technology, Chinese Academy of Agriculture Sciences, Beijing, China; ^2^Wageningen Food and Biobased Research, Wageningen University and Research, Wageningen, Netherlands; ^3^Laboratory of Food Chemistry, Wageningen University, Wageningen, Netherlands

**Keywords:** immunomodulatory proteins, FIPs, glycosylation, immunomodulaion, hemagglutination, structure-function relationship

## Abstract

Fungal immunomodulatory proteins (FIPs) are a group of proteins found in fungi, which are extensively studied for their immunomodulatory activity. Currently, more than 38 types of FIPs have been described. Based on their conserved structure and protein identity, FIPs can be classified into five subgroups: Fve-type FIPs (Pfam PF09259), Cerato-type FIPs (Pfam PF07249), PCP-like FIPs, TFP-like FIPs, and unclassified FIPs. Among the five subgroups, Fve-type FIPs are the most studied for their hemagglutinating, immunomodulating, and anti-cancer properties. In general, these small proteins consist of 110–125 amino acids, with a molecular weight of ~13 kDa. The other four subgroups are relatively less studied, but also show a noticeable influence on immune cells. In this review, we summarized the protein modifications, 3-dimensional structures and bioactivities of all types of FIPs. Moreover, structure-function relationship of FIPs has been discussed, including relationship between carbohydrate binding module and hemagglutination, correlation of oligomerization and cytokine induction, relevance of glycosylation and lymphocyte activation. This summary and discussion may help gain comprehensive understanding of FIPs' working mechanisms and scope future studies.

## Introduction

Fungi can potentially contain many bioactive proteins, including lectins, ribosome-inactivating proteins, laccases, nucleases, glycoproteins/glycopeptides, and immunomodulatory proteins (1–3). Fungal immunomodulatory proteins (FIPs) are a group of proteins found in fungi, which show noticeable immunomodulatory activity (4, 5). In recent decades, they have been widely studied for their pharmaceutical utilizations (2, 3). Ling-Zhi 8 (LZ-8) is the first FIP discovered in *Ganoderma lucidum* (6). Till now, more than 38 types of FIPs have been identified (see Table 1).

**Table 1:** Summary of FIPs and classification.

**Classification**	**Name**	**Accession**	**Source**	**References**
Fve-type FIPs	rFIP-bbo	KDQ10166.1	*Botryobasidium botryosum*	(7)
	rFIP-cru	AKU37620.1	*Chroogomphis rutilus*	(8)
	rFIP-dsp2	XP_007363541	*Dichomitus squalens*	(9)
	FIP-fve	P80412.1	*Flammulina velutipe*	(4)
	rFIP-gap1	AEP68179.1	*Ganoderma applanatum*	(10)
	rFIP-gap2	ART88472.1	*Ganoderma applanatum*	(10)
	rFIP-gat	AJD79556.1	*Ganoderma atrum*	(11)
	LZ-8	P14945.2	*Ganoderma lucidum* (Ling Zhi)	(6)
	rLZ-9	na	*Ganoderma lucidum*	(12)
	rFIP-gmi	3KCW	*Ganoderma microsporum*	(13)
	rFIP-gsi	na	*Ganoderma sinensis*	(14–16)
	FIP-gts	na	*Ganoderma tsugae*	(17)
	rFIP-SN15	na	Intergeneric shuffled library	(18)
	rFIP-SJ75	na	Intergeneric shuffled library	(19)
	rFIP-lrh	na	*Lignosus rhinocerotis*	(20)
	rFIP-lti1	na	*Lentinus tigrinus*	(21)
	rFIP-lti2	na	*Lentinus tigrinus*	(21)
	rFIP-nha	EEU37941.1	*Nectria haemotococca*	(12)
	rFIP-ppl	AJL35148.1	*Postia placenta*	(9)
	rFIP-sch2	AQQ80204.1	*Stachybotrys chlorohalonata*	(22)
	rFIP-sch3	KEY70185.1	*Stachybotrys chlorohalonata*	(22)
	rFIP-tvc	na	*Trametes versicolor*	(23)
	FIP-vvo	na	*Volvariella volvacea*	(24)
	rFIP-vvo77	na	*Volvariella volvacea*	(25)
	rFIP-vvo78	na	*Volvariella volvacea*	(25)
	rFIP-vvo79	na	*Volvariella volvacea*	(25)
	rFIP-vvo80	na	*Volvariella volvacea*	(25)
	rFIP-vvo82	na	*Volvariella volvacea*	(25)
	rFIP-vvo98	na	*Volvariella volvacea*	(25)
Cerato-type FIPs	ACA	AAT11911.1	*Antrodia camphorata*	(26)
	YZP	AGH06133.1	*Trametes versicolor (Yunzhi)*	(27)
PCP-like FIPs	PCP	AEM91639.1	*Poria cocos*	(28)
TFP-like FIPs	TFP	ABL96299.1	*Tremella fuciformis*	(29)
Unclassified FIPs	APP	na	*Auricularia polytricha*	(30)
	HEP3	na	*Hericium erinaceus*	(31)
	PCiP	na	*Pleurotus citrinopileatus*	(32)
	PEP 1b	na	*Pleurotus eryngii*	(33)
	TVC	na	*Trametes versicolor*	(34)

*na, not available*.

**Figure 1:**
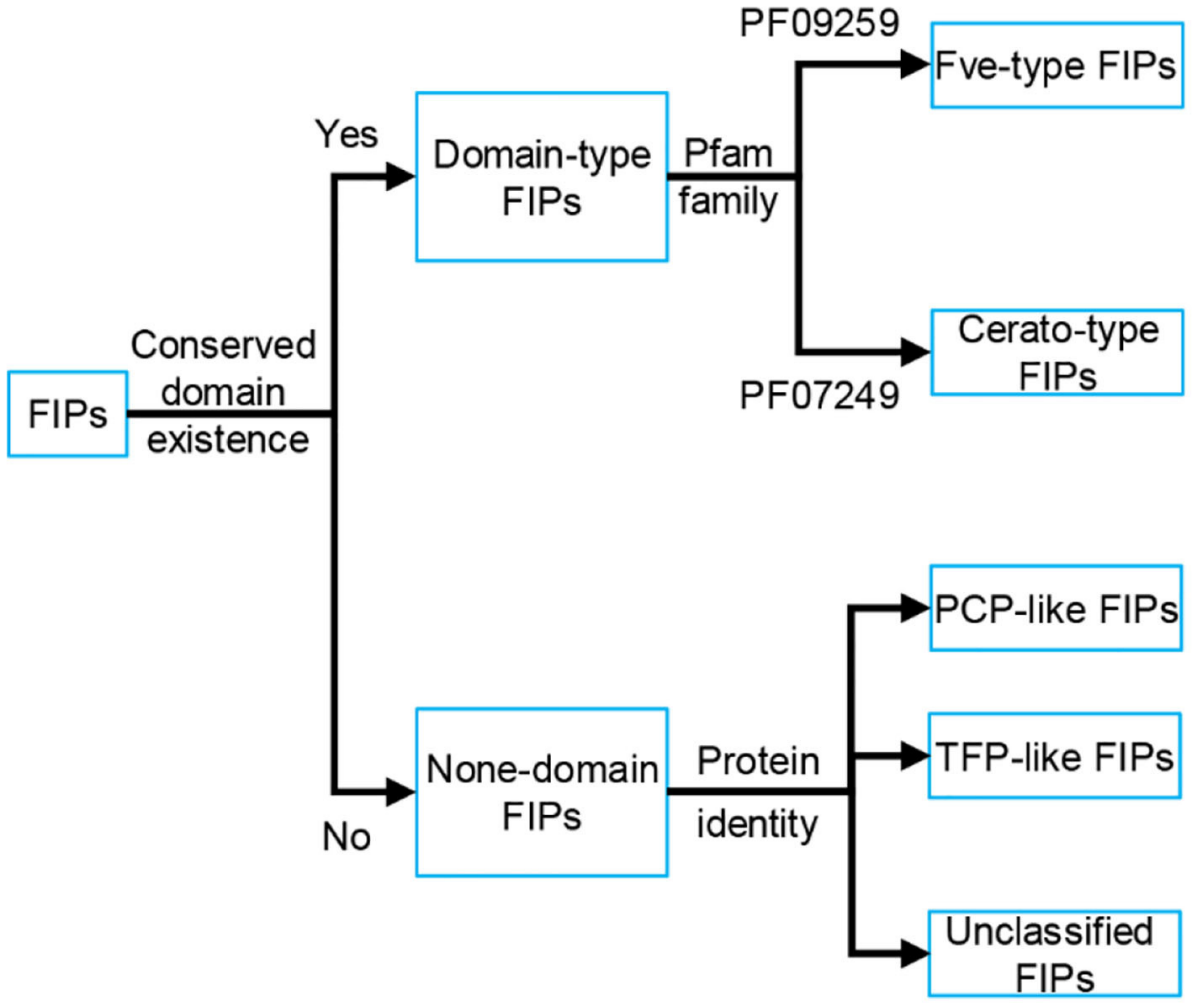
Classification of FIPs based on the conserved structure and protein identity. Depending on the existence of conserved domains, FIPs could be classified into domain-type FIPs and none-domain FIPs. In domain-type FIPs, Fve-type FIPs have the assigned Pfam Fve family domain signature PF09259, while Cerato-type FIPs contain the Pfam PF07249 domain signature. In none-domain FIPs, FIPs were separated into PCP-like FIPs, TFP-like FIPs and unclassified FIPs based on protein identity. As the amino acid sequence of unclassified FIPs has not yet been elucidated, they could not be classified and have been placed separately.

Based on the conserved structure and protein identity (see Figure 1), FIPs can be classified into 5 subgroups: Fve-type FIPs, Cerato-type FIPs, PCP-like FIPs, TFP-like FIPs, and unclassified FIPs (see Figure 1 and Table 1). Among the five subgroups, Fve-type FIPs are the largest subgroup with 29 members which have the assigned Pfam Fve family domain signature PF09259. *Antrodia camphorate* immunomodulatory protein (ACA) and *Trametes versicolor* “Yun-Zhi” protein (YZP) belong to the second subgroup, named Cerato-type FIPs, which contains the Pfam PF07249 domain. The third subgroup is formed by the PCP-like FIPs with only one member till now, *Poria cocos* immunomodulatory protein (PCP). Besides PCP, three kinds of hypothetical proteins from *Wolfiporia cocos* (Accession PCH39154.1, PCH35963.1, and PCH34729.1) might also belong to this subgroup. Currently, the fourth subgroup just contains *Tremella fuciformis* protein (TFP), with no other high-identity proteins having been identified by BLAST. This group is indicated as TFP-like FIPs. The other FIPs could not be classified since their amino acid sequence has not yet been elucidated. Hence, they are categorized as unclassified FIPs, including *Auricularia polytricha* immunomodulatory protein (APP), *Pleurotus citrinopileatus* immunomodulatory protein (PCiP), the immunomodulatory protein from *T. versicolor* (named TVC), the immunomodulatory protein from *Hericium erinaceus* (named HEP3), and the immunomodulatory protein from *Pleurotus eryngii* (named PEP 1b).

In general, Fve-type FIPs consist of 110–125 amino acids, with a molecular weight of ~13 kDa. This subgroup of FIPs was mainly studied for their hemagglutinating, immunomodulating, and anti-cancer properties, which have been reviewed in several articles. These reviews mostly focused on the their bioactivities, heterologous production, physicochemical properties, and proposed molecular anti-tumor mechanisms (5, 35–37). However, the mechanism behind their immunomodulatory activity needs to be updated, and the detailed information about their protein structure and function relationship have not yet been reviewed. Proteins belonging to the other four FIP subgroups have shown a noticeable influence on immune cells. However, they were neither extensively studied, nor critically discussed. In this review, we have summarized protein modifications like glycosylation, the 3-dimensional structure, and bioactivity of all types of FIPs, and try to find relationships between structural features and immunomodulatory activity, in order to gain a better understanding of the mechanism of FIPs' bioactivity and scope further research.

## Structure Analysis

### Structure of FIPs

**Figure 2:**
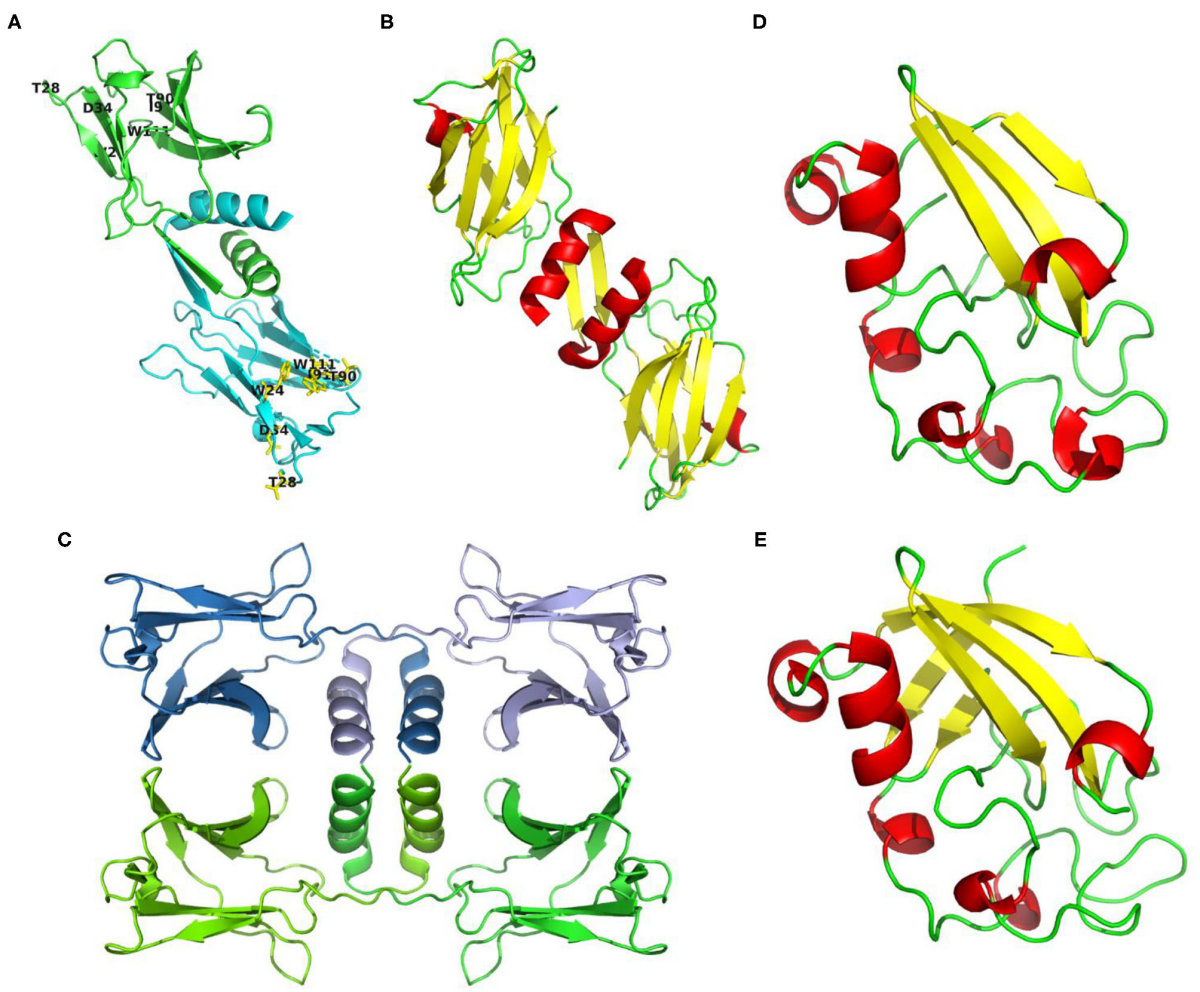
Structures or structure predictions of FIPs. **(A)** FIP-fve (PDB code: 1OSY) colored by monomer in cyan and green, and labeled with purposed CBM34-like structure in yellow (W24, T28, D34, T90, I91, and W111 of FIP-fve); **(B)** LZ-8 (PDB code: 3F3H) colored by secondary structure (helixes in red, sheets in yellow, loops in green); **(C)** FIP-gmi (PDB code: 3KCW) colored by monomer in skyblue, lightblue, chartreuse, and green; **(D)** Structure prediction of ACA based on 3m3g.1. via SWISS-MODEL (38–42), and colored by secondary structure (helixes in red, sheets in yellow, loops in green); **(E)** Structure prediction of YZP based on 3suk.1.A via SWISS-MODEL (38–42), and colored by secondary structure (helixes in red, sheets in yellow, loops in green).

In general, the amino acid sequence of reported Fve-type FIPs is rich in valine and aspartic acid, and short of cysteine and histidine. Based on the circular dichroism spectrum analysis and prediction of ExPASy, they consist of 1-3 α-helixes, 7-9 β-sheets, and some random coils (see Supplementary Table 1). The 3-dimensional structures of three FIPs have been determined via X-ray diffraction in earlier studies: FIP-fve (PDB code: 1OSY), LZ-8 (PDB code: 3F3H) and FIP-gmi (PDB code: 3KCW) (see Figures 2A–C). The structure showed that monomers of Fve-type FIPs consist of two parts, the N-terminal and C-terminal domains. The N-terminal domain starts with an N-terminal α-helix, which is an essential structure for all Fve-type FIPs. The α-helix is followed by a β-sheet (1OSY and 3F3H), which is essential for the dimeric structure, or a stretch of random coil (3KCW). The C-terminal domain is a sandwich-type Fibronectin III domain, which mainly consists of 7 β-sheets (13, 43, 44). The monomer of Fve-type FIPs assembles into a homodimeric (1OSY and 3F3H) or tetrameric structure (3KCW) via non-covalent interaction, such as hydrophobic interactions and hydrogen bonds. The dimerization sustained by domain swapping (45, 46) mainly depends on the N-terminal α-helix and β-sheet (43, 44), while there is no detailed information about the dynamics of tetramer formation. Notably, after the N-terminal α-helix in FIP-gmi (3KCW), there is a stretch of random coil, instead of a β-sheet. This difference may be relevant for the occurrence of the different oligomeric states, while further study is needed to clarify the underlying mechanism.

Till now, only the amino acid sequence of ACA, YZP, PCP, and TFP has been identified, and no primary structure information is available about unclassified FIPs. Though no amino acid sequence is known, some articles report the amino acid composition. For instance, APP is relatively rich in threonine (11.5%), but contains no histidine, little methionine (1.05%) and cysteine (1.08%) (30). Besides, PCiP has a high leucine content (10.76%), but little cysteine (0.56%), and methionine (1.22%) (32). We predicted their secondary structure via ExPASy based on the known sequence (see Supplementary Table 1). Protein structures of FIPs at crystal level or relevant NMR data for proteins from other subgroups have not been provided. A structure prediction of Cerato-type FIPs could be obtained via SWISS-MODEL using 3m3g.1.A and 3suk.1.A (Cerato-platanin proteins) as modeling template for ACA and YZP, respectively (see Figures 2D,E) (38–42). Cerato-platanin proteins are small, secreted proteins involved in the various stages of the host-fungus interaction process, acting as phytotoxins, elicitors, and allergens (47, 48). Modeled ACA and YZP show a spheroid-like structure, formed by short α-helixes, three (ACA) or five β-sheets (YZP), and lots of random coils (see Figures 2D,E). The three β-sheets of ACA exits in one layer, while the five β-sheets of YZP fold into two perpendicular layers, which may lead to a more stable core.

### Glycosylation Modification of FIPs

Protein glycosylation is an important post-translational modification that may influence protein structure and function in eukaryotes (49–52). Till now, we found that 8 native or heterologous expressed specimens of FIPs could be glycosylated, and 12 specimens of FIPs have potential glycosylation sites.

As shown in Table 2, several Fve-type FIPs have potential N-glycosylation sites, and some of them were glycosylated in heterologous expression systems. In 2013, Bastiaan-Net et al. expressed recombinant FIP-fve in *Picha pastoris*, which resulted in three bands on SDS-PAGE gel, representing the non-glycosylated, single glycosylated and double glycosylated FIP-fve respectively, demonstrating that both predicted glycosylation sites (N36 and N54) could be glycosylated. Whether these results correspond to the glycosylation state of native FIP-fve could not be concluded with certainty, as proteins will be over-glycosylated in the yeast expression systems (52). Interestingly, rFIP-gts expressed in Sf21 cells can be produced in glycosylated (relative molecular mass (Mr) ~15 kDa) as well as in non-glycosylated forms (Mr ~13.5 kDa), which was confirmed by glycoprotein staining. Specifically, the glycosylated rFIP-gts was guided by a signal peptide from bombyxin (SP_bbx_; the recombinant vector vAcP10SP_bbx_Gts), while the rFIP-gts without this signal peptide (the recombinant vector vAcP10Gts) existed as a non-glycosylated form. Wu et al. thought that the signal peptide (SP_bbx_) upstream of rFIP-gts facilitated entry into the endoplasmic reticulum where the glycosylation process takes place (8). However, no putative glycosylation site was demonstrated in the article, nor was it predicted by the NetNGlyc 1.0 Server and NetOGlyc 4.0 Server.

**Table 2:** Glycosylation modification of FIPs.

**FIPs**	**Glycosylation**	**Native/heterologous expression/predicted**	**References**
ACA	N-glycosylated protein	Native	(26)
PCP	Disulfide-linked heterodimeric glycoprotein with N- and O-glycosylation.	Native	(28)
rFIP-gts	Can be glycosylated when expressed by Sf21 insect cells	Sf21 insect cells	(8)
rFIP-fve	Two N-glycosylated sites: N36 and N54	*P. pastoris*	(12)
rFIP-nha	Two N-glycosylated sites: N5 and N39	*P. pastoris*	(12)
rFIP-cru	One confirmed and one potential glycosylation sites: N29 (79.64%^*^) and N36 (76.59%^*^)	*P. pastoris*	(53)
rFIP-gap1	One N-glycosylated sites: N38	*P. pastoris*	(10)
rFIP-gap2	Two N-glycosylated sites: N31 (79.49%^*^) and N38 (76.40%^*^)	*P. pastoris*	(10)
FIP-lrh	Four potential O-linked glycosylation: S1, T4, T6, and S58	Predicted^#^	(20)
rFIP-gsi	One potential N-glycosylated site: N36 (74.31%)	Predicted^*^	(15, 16)
FIP-lti1	Two potential glycosylation sites: N27 (61.63%^*^) and N34 (57.98%^*^)	Predicted^*^	(21)
FIP-lti2	Two potential glycosylation sites: N30 (81.12%^*^) and N37 (61.80%^*^)	Predicted^*^	(21)
FIP-SN15	Two potential glycosylation sites: N29 (80.11%^*^) and N36 (74.30%^*^)	Predicted^*^	(18)
FIP-SJ75	Two potential glycosylation sites: N31 (76.10%^*^) and S34 (50%^*^)	Predicted^*^	(19)
FIP-dsp2	Two potential glycosylation sites: N36 (66.49%) and S32 (58.80%)	Predicted^*^	(22)
FIP-gat	Two potential glycosylation sites: N29 (80.10%) and N36 (74.29%)	Predicted^*^	(11)
FIP-gmi	One potential glycosylation sites: S32 (64.29%)	Predicted^*^	(54)
FIP-gsi	Two potential glycosylation sites: N29 (80.11%) and N36 (74.31%)	Predicted^*^	(15)
FIP-sch2	One potential glycosylation sites: S33 (50.90%)	Predicted^*^	(22)
FIP-sch3	One potential glycosylation sites: S33 (53.04%)	Predicted^*^	(22)

* *Predicted by NetNGlyc 1.0 (http://www.cbs.dtu.dk/services/NetNGlyc/) and NetOGlyc 4.0 (http://www.cbs.dtu.dk/services/NetOGlyc/)*.

# *Means FIP-lrh has no positive O-glycan site predicted by NetOGlyc 4.0 (http://www.cbs.dtu.dk/services/NetOGlyc/), but Pushparajah et al. obtained the results by NetOGlyc 4.0 and GlycoEP (http://www.imtech.res.in/raghava/glycoep/) (20)*.

Also, native ACA and PCP are glycoproteins (26, 28). ACA is predominately an N-glycosylated protein, as deglycosylated ACA (treated with N-glycosidase F, a specific N-glycosylation protease) shows the same molecular weight as rACA expressed by *Escherichia coli*. Predicted by GlyNGly 1.0 Service, asparagine at residue 20 (N20) of the full-length ACA (16 kDa) had the highest possibility (80.53%) to be N-glycosylated, followed by N31 and N87 (with 63.51 and 66.68% possibility, respectively) (26). Native PCP (35.6 kDa) is a disulfide-linked heterodimeric glycoprotein consisting of 14.3 and 21.3 kDa subunits with N- and O-glycosylation which contributes ~7 kDa to its molecular mass (28). Interestingly, there is only one predicted N-glycosylation site (N142, with 52.90% possibility) found by the NetNGlyc 1.0 and NetOGlyc 4.0 Servers. Meanwhile, there is some controversy as to the composition of PCP. Lu et al. thought PCP to be composed of two identical 12.8 kDa protein subunits (73 residues cleaved); however, these subunits would be asymmetrically modified by N- and O- glycosylation (55). Still, PCP was not heterologous expressed to verify such hypothesis. In contrast, Li et al. expressed rPCP (19 residues cleaved as signal peptide) in *P. pastoris* system, and showed that rPCP existed as two bands (Mr ~18 and ~20 kDa) under reducing condition and one band (Mr ~37 kDa) under non-reducing condition. Besides, rPCP was only N-glycosylated, as confirmed by PNGase F (specific for N-glycans) treatment (56). Though the rPCP had to some extent a similar bioactivity to native PCP, such as upregulation of interleukin (IL)-1β and tumor necrosis factor (TNF)-α gene expression next to induction of macrophage TNF-α secretion (28, 55, 56), it's utilization to replace native PCP still need consideration.

## Health-Promoting Activities of FIPs

As the antitumor activity of FIPs has been described clearly in recent reviews (36, 37), we focus here specifically on immunomodulatory activity and hemagglutination properties.

### Immunomodulatory Activity of Fve-Type FIPs

**Figure 3:**
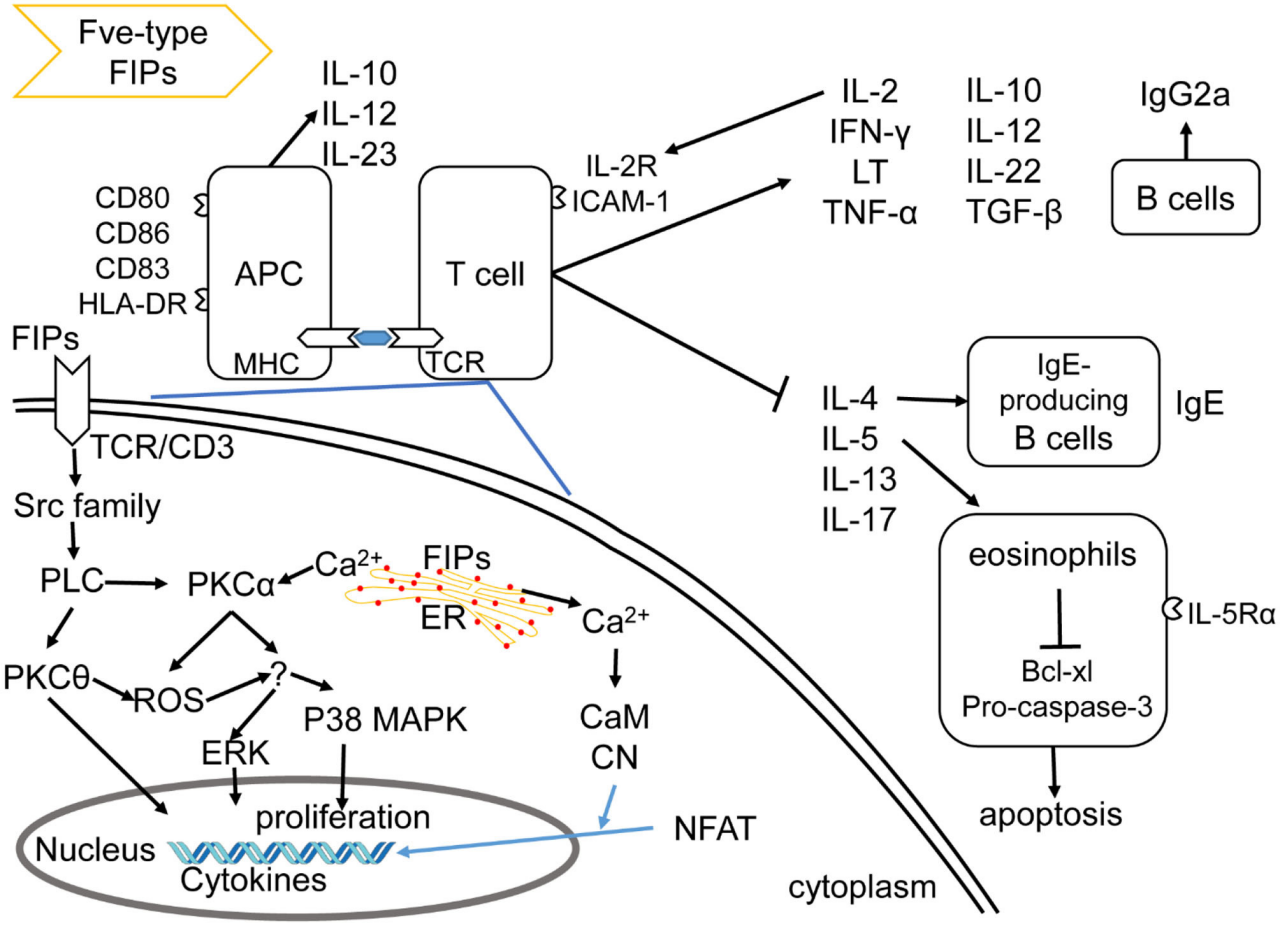
Effects of Fve-type FIPs on the immune system, adapted from Hsu et al. (57). Fve-type could enhance expression of cell adhesion molecules and regulate secretion of cytokines and chemokines on APCs (including DCs and macrophage) and T cells, which could also contribute to APCs and T cell reaction. Fve-type FIPs could up-regulate IL-2, IFN-γ, LT, TNF-α, IL-10, IL-12, IL-22, TGF-β, and down-regulate IL-4, IL-5, IL-13, IL-17 in T cells, which may contribute to anti-allergic and anti-inflammatory effects in combination with IgE downregulation, eosinophil inhibition and IgG2a induction. Mechanistically, T cell cytokine production (IL-2 and IFN-γ) has been described in the down-left part, including activation of signaling pathway and Ca^2+^ flow.

Fve-type FIPs can regulate cellular and humoral immunity (Figure 3), which includes activation of immune cells, leading to immune-mediated anti-allergic, anti-inflammatory, and anti-tumor effects.

#### Activation of Immune Cells

Fve-type FIPs activate immune cells via inducing secretion of cytokines and chemokines, increasing expression of cell adhesion molecules, as well as enhancing lymphocyte proliferation. As shown in Table 3, FIPs behave distinctively in activation of immune cells. For instance, FIP-vvo and FIP-gsi could induce transcriptional expression of IL-4, TNF-α, and lymphotoxin (LT) (14, 24), whereas no significant regulation of IL-4 secretion was reported following treatment with LZ-8 (58) or FIP-fve (64). However, most FIPs could proliferate mouse splenocytes and human peripheral blood lymphocytes (hPBLs), as well as induce IL-2, interferon (IFN)-γ, and IL-2 receptor (IL-2R) transcriptional expression.

**Table 3:** Activation of immune cells induced by Fve-type FIPs.

**FIPs**	**Induction of cell proliferation**	**Cytokines secretion**	**Cytokines expression**	**Cell adhesion molecules**	**References**
LZ-8	Mouse splenocytes; hPBLs; T cell (monocyte dependent); hPBMCs	IFN-γ^↑^, TNF-α^↑^, IL-1β^↑^, IL-2^↑^, IL-12 p40^↑^, IL-12p70^↑^, IL-23^↑^, IL-4^N^	CCL2^↑^, IL-10^↓^, CXCL10^↓^, IL-6^N^	CD25^+^(T cells)^↑^; IL-2R (T cells)^↑^; ICAM-1 (PBLs)^↑^; CD54 (T cells)^↑^; HLA-DR (DCs)^↑^, CD80 (DCs)^↑^, CD86 (DCs)^↑^, CD83 (DCs)^↑^, CD44 (T cells)^↑^, CD154 (T cells)^↑^, CD86 (macrophages)^↑^; MHCII (macrophages)^↑^	(57–63)
FIP-fve	hPBLs (G1/G0 to S)	IL-2^↑^, IFN-γ^↑^, IL-10^↑^, TGF-β^↑^, IL-22^↑^, IL-4^N^, IL-5^↓^, IL-6^↓^, IL-13^↓^, IL-17^↓^		ICAM-1 (hPBMCs)^↑^, IL-5R (eosinophils)^↓^, CD95 (eosinophils)^↑^, MHCI (PBMCs)^↑^, MHCII (PBMCs)^↑^, CD80 (PBMCs)^↑^	(4, 64–71)
FIP-vvo	hPBLs		IL-2^↑^, IL-4^↑^, IFN-γ^↑^, TNF-α^↑^, LT^↑^, IL-1^N^, IL-3^N^, IL-5^N^, IL-6^N^	IL-2R (mouse splenocytes)^↑^	(24)
FIP-gsi			IL-2^↑^, IL-3^↑^, IL-4^↑^, IFN-γ^↑^, TNF-α^↑^, IL-1α^N^, IL-5^N^, IL-6^N^, LT^N^	IL-2R (mouse splenocytes)^↑^	(14)
FIP-cru		IL-2^↑^			(53)
FIP-gaps	Mouse splenocytes	IL-2^↑^, IFN-γ^↑^			(10)
FIP-SJ75	RAW264.7 cells		TNF-α^↑^, IL-6^↑^, IL-10^↑^, TGF-β1^↑^,		(19)
FIP-ltis			IL-1β^↓^, IL-6^↓^, TNF-α^↓^	CD4^+^(T cells)^↑^; CD25^+^(T cells)^↑^	(21)
FIP-nha	Mouse spleen lymphocyte	IL-2^↑^			(72)
FIP-ppl	Mouse splenocytes	IL-2^↑^			(9)
FIP-tvc			IL-1α^↑^, IL-2^↑^, IL-5^↑^, IL-6^↑^, TNF-α^↑^, LT^↑^, IL-3^N^, IL-4^N^, IFN-γ^N^	IL-2R (mouse splenocytes)^N^	(23)

↑ *In the superscript means up-regulation*.

↓ *In the superscript means down-regulation*.

N *In the superscript means no significant change*.

*(Cells) in which the adhesion molecules were detected*.

LZ-8 is one of the most studied FIPs for immune modulation effects. It could induce mitogenesis toward mouse splenocytes (59), hPBLs (60), human mixed lymphocyte (purified T cells and allogeneic B cells) (61). Its behavior on hPBLs is monocyte-dependent, similar to phytohemagglutinin and other lectin mitogens (60) which can activate T cells and Dendritic cells (DCs), and induces the production of quite a few cytokines and cell adhesion molecules.

There is some insight into the mechanism of LZ-8 in regulating IL-2 transcriptional expression in T cells. As shown in Figure 3, the rLZ-8-mediated signal-transduction pathways such as protein tyrosine kinase (PTK)/protein kinase C (PKC)/reactive oxygen species (ROS), PTK/phospholipase C (PLC)/PKCα/extracellular signal-regulated kinase (ERK)1/2, and PTK/PLC/PKCα/p38, could up-regulate IL-2 transcriptional expression in T cells (57). Meanwhile, TCR/cluster of differentiation (CD)3 complex is one of the putative binding sites of, or receptors for, rLZ-8 in T cell activation (57). With respect to the IL-2 secretion, inhibition of PKC, Ca^2+^ influx, mitogen-activated protein kinase kinase (MEK)1/ERK1/2 pathway, or Src-family kinases could result in a significant reduction and inhibition of IL-2 secretion (upon incubation with rLZ-8). These evidences indicate the mentioned signaling pathways and Ca^2+^ influx to be important for the rLZ-8-mediated IL-2 secretion by T cells (57).

Most likely, LZ-8 is also able to activate antigen-presenting cells (APCs). Specifically, Lin et al. (58) demonstrated the ability of LZ-8 to induce activation and maturation of DCs (58). In addition, Li et al. (62) suggested that LZ-8 activates the PI3K/Akt and MAPK signaling pathways after internalization by macrophages. However, the exact underlying mechanism of DCs (58, 73) and macrophages (62) activation by LZ-8 still needs to be further explored, especially its supposed TLR4 binding, since minor lipopolysaccharide (LPS) contamination could cause similar results in DCs and macrophages (74, 75). FIP-SJ75, which is a chimera composed of LZ-8, FIP-fve and FIP-vvo (19), triggered similar cytokine expression profiles in macrophages as seen for LZ-8 (shown in Table 3). Its underlying mechanism was still unknown, which needs further investigation.

FIP-fve is also a potent T-cell activator, mediating its effects via cytokine regulation of p38 mitogen-activated protein kinase (MAPK), especially IFN-γ secretion (64). Besides, FIP-fve stimulates IFN-γ transcriptional expression in human peripheral blood mononuclear cells (hPBMCs) via the modulation of Ca^2+^ release and the activation of PKCα (65). Notably, p38 MAPK activation, PKC pathways, and Ca^2+^ flow are vital in both FIP-fve and LZ-8 mediated cytokine transcriptional expression (57, 64). FIP- fve initially induces Ca^2+^ release which results in facilitating the activation of Ca^2+^-dependent PKC-α (65), while the mechanism underlying Ca^2+^ influx in LZ-8 treated T cells is not known (57). Meanwhile, both FIP-fve and LZ-8 can aggregate hPBLs and consequently increase the intercellular cell adhesion molecule (ICAM)-1 expression, which is associated with increasing IFN-γ, TNF-α, IL-1β secretion levels (30, 60), and may contribute to their immunomodulation properties.

#### Anti-allergic and Anti-inflammation Effects

Fve-type FIPs have been studied for their anti-allergic and anti-inflammatory properties for quite a few years. These studies focus on asthma, airway inflammation, food allergy, systemic anaphylaxis reactions, and other graft-tolerance or inflammation reactions.

Allergic asthma is a chronic airway inflammation, which would cause activation of CD4^+^ T cell, eosinophils, and IgE-producing B cells, polarize T helper (Th)2 cells, as well as induce Th2 cytokines (such as IL-4, IL-5, IL-13) and secretion of other cytokines (IL-17, IL-33, IL-25) (76, 77). FIP-fve could suppress allergen-induced asthma, airway inflammation (66, 67, 78, 79), respiratory syncytial virus (RSV) replication, RSV-induced inflammation (80), as well as eosinophil-related allergic inflammation *in vitro* or *in vivo* (68). More specifically, in allergen-induced asthma or airway inflammation mice models, both pre-treatment and post-treatment with orally administrated FIP-fve suppressed the airway hyperresponsiveness in allergen-sensitized mice (66, 79). Upon methacholine challenge, significantly decreased the number of infiltrating inflammatory cells (neutrophils and eosinophils) as well as secretion of IL-17 and Th2 cytokines (IL-4, IL-5, IL-13), and increased Th1 cytokines (IFN-γ, IL-10, IL-12, IL-22, TGF-β) production in bronchoalveolar lavage fluid and serum (66, 67, 79). In addition, allergen-specific immunoglobulin IgE in serum was significantly decreased, whereas the serum IgG2a level increased significantly (66, 67, 79). Even the structural changes of inflammatory lung tissue would become nearly normal via oral FIP-fve treatment based on lung histopathological studies (66). Regarding RSV-related disease, pre-treatment with FIP-fve inhibited RSV replication after 24 h infection *in vitro*, and oral administered FIP-fve decreased RSV-induced airway hyperresponsiveness, airway inflammation, and IL-6 expression in bronchoalveolar lavage fluid of RSV-infected mice (80). What's more, in eosinophil-related allergic inflammation, FIP-fve can inhibit IL-5-mediated survival of eosinophils through down-regulating IL-5Rα expression on the cells' surface, and enhancing eosinophil apoptosis via down-regulation of BCL-XL [a pro-survival protein in apoptosis regulating BCL-2 protein family (81)] and pro-caspase 3 expressions *in vitro* (68). In summary, oral FIP-fve treatment in mice is described to exert beneficial effects on both airway allergic or inflammatory symptoms, as well as on secondary makers such as secreted cytokines, except for eosinophil-related allergic inflammation.

Hsieh et al. intraperitoneally injected ovalbumin to mimic food allergy in a murine model. Oral administration of FIP-fve during allergen sensitization upregulated IFN-γ and downregulated IL-5 expressions in splenocytes, decreased ovalbumin-specific IgE response, and enhanced IgG2a response, which is similar to airway inflammation (82). Besides, it could protect the mice from systemic anaphylaxis-like symptoms after subsequent oral challenge with the same allergen (82).

Allergic asthma, IgE mediated food allergy and symmetric anaphylaxis reaction are all type I hypersensitivity reactions involving antibody-mediated immune cell responses to an allergen (83). However, the Arthus reaction is a type III hypersensitivity reaction that is immune complex-mediated, and involves the deposition of antigen/antibody complexes in the vascular walls (83). LZ-8 and FIP-fve could suppress bovine serum albumin-induced Arthus reaction and systemic anaphylaxis reaction in mice, which was explained by reduction of antibody production (4, 84). Fip-vvo significantly reduced the production of bovine serum albumin-induced Arthus reaction in mice *in vivo*, whereas there was no apparent effect in the prevention of systemic type I anaphylaxis reactions (24). Mechanistically, FIP-vvo induced mostly Th1-specific transcriptional expression of cytokines (IL-2, IFN-γ, and LT), next to the transcription of Th2-specific IL-4 (within 4 h) in mouse spleen cells (24), while there is no information about the secretion of cytokines. In conclusion, the immunomodulatory activity of FIP-vvo may, to some extent, be lower than FIP-fve or LZ-8.

Other benefits of FIPs include suppression of local swelling of mouse footpads by FIP-fve (4). LZ-8 suppressed effects in allogeneic tissue transplantation without side effects (61), helped increase immunity on leukopenia (low white blood cell count) induced by cyclophosphamide in mice (85), and improved both non-alcoholic fatty liver disease and early atherogenesis (fat- deposits in the arteries) because of its anti-inflammatory effect (86). FIP-lti1 and FIP-lti2 have been shown to mitigate ConA-induced liver oxidative injury (21). FIP-gmi was recently found to exert anti-inflammatory effects on neuron/glia cells (87), human fibrotic buccal mucosal fibroblasts (88), as well as intestinal mucosa and the tongue (89).

#### As Tumor Vaccine or Adjuvant

LZ-8 may be a promising adjuvant to enhance the efficacy of DNA vaccines by activating DCs. DCs activation could induce antigen-specific T cell activation, which contributed to Th1 and cytotoxic T lymphocyte responses induced by the vaccine against mouse bladder tumor (73). FIP-fve might help tumor immunotherapy via both innate and adaptive immunity. CD4^+^ T cells, CD8^+^ T cells, and IFN-γ play critical roles in conferring the anti-tumor effects. More specifically, co-immunized mice by tumor antigen and FIP-fve showed induction of antigen-specific antibodies and increased the expansion of antigen-specific IFN-γ-producing CD4^+^ T cells and CD8^+^ T cells, leading to an enhanced antigen-specific humoral and cellular type 1 anti-tumor immune response (90). Oral administration of FIP-fve significantly increased the tumoricidal capacity of peritoneal macrophages and tumor-specific splenocytes, and up-regulated the expression levels of MHC class I and II molecules and costimulatory molecule CD80 on peripheral blood mononuclear cell to inhibit tumor growth and angiogenesis (91). Oral treatment of FIP-fve did not influence body weight, and the tumoricidal effect of FIP-fve was significantly decreased when the mice were co-injected with IFN-γ neutralization, confirming that FIP-fve exerts its function via immune, rather than via a cytostatic mechanism (91).

### Immunomodulatory Activity of Other Subgroups of FIPs

**Figure 4:**
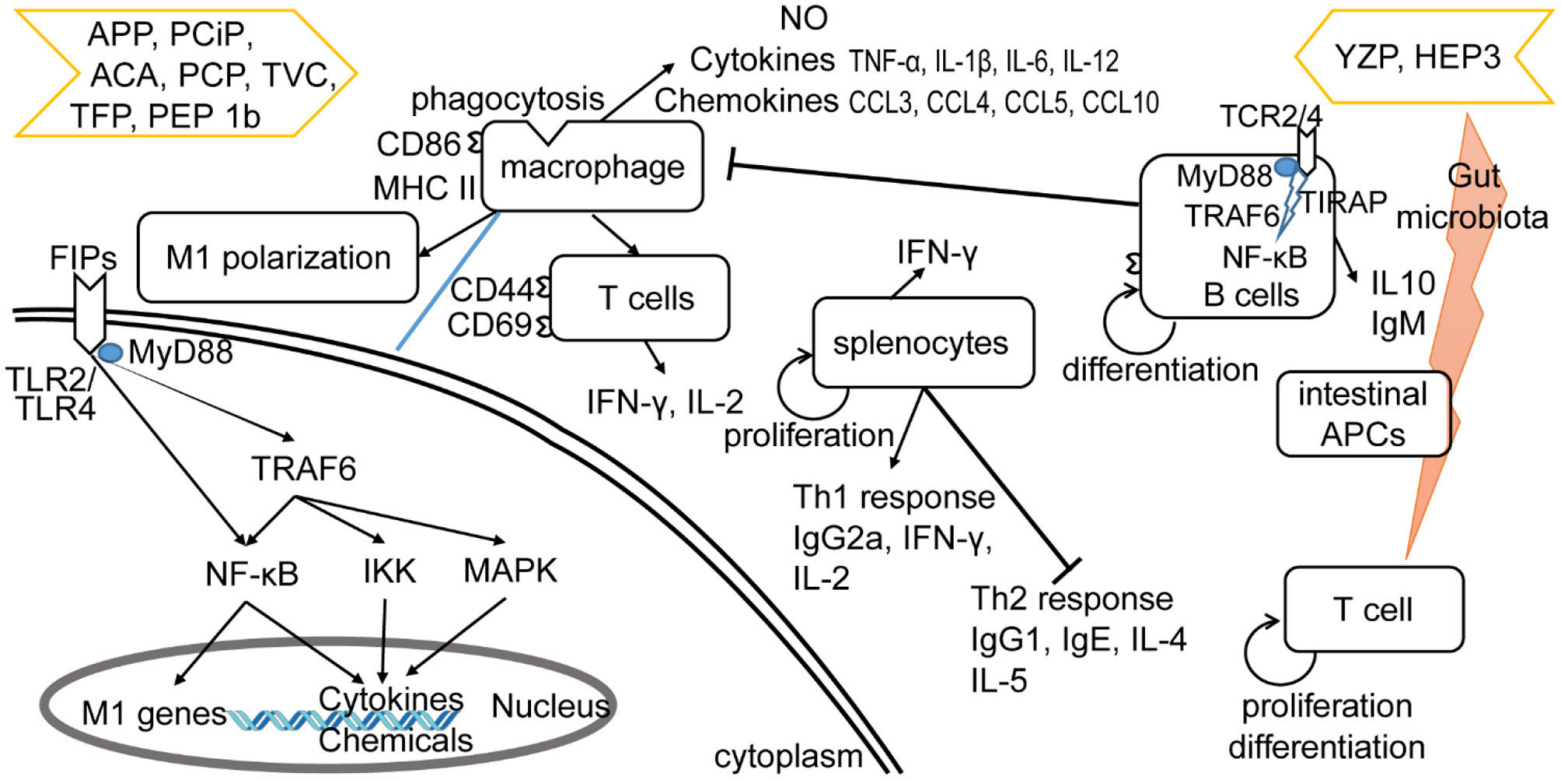
Effects of other type of FIPs on the immune system. FIPs mentioned on the left side (APP, PCiP, ACA, PCP, TVC, TFP, and PEP 1b) induce an LPS-mimicking proinflammatory response or enhance the response. More specific, FIPs in the left side could activate macrophages, T cells, and splenocytes. The mechanism of macrophage activation has been shown in down-left part. FIPs in the right side (YZP and HEP3) could reduce activation of LPS-activated macrophages, suppress inflammatory cytokine expression, and show other anti-inflammatory and anti-tumor effects via B cell regulation (YZP) or gut microbiota (HEP3).

The other four subgroups of FIPs were identified to modulate macrophage activity by regulating cytokine and chemokine production, activating signaling pathways (28, 29, 33), and/or M1 (classically activated macrophages) polarization (see Table 4). Besides, some of them could activate lymphocytes or enhance lymphocytes activation (see Table 4). As shown in Figure 4, FIPs mentioned in the yellow arrow on the left side (APP, PCiP, ACA, PCP, TVC, TFP, and PEP 1b) can induce an LPS-mimicking pro-inflammatory response or enhance the response, while others (YZP and HEP3; right-hand side) could suppress LPS-induced responses (similar with Fve-type FIPs) or inhibit tumor growth indirectly via gut microbiota. However, most research used native FIPs extracted from fungi, and in some cases there was no check for other immunomodulatory compounds (especially endotoxin contamination) reported, which may have biased outcomes.

**Table 4:** Activation of immune cells induced by other subgroups of FIPs.

**FIPs**	**Cells modulation**	**NO induction**	**Cytokines/chemokines secretion**	**Cytokines/chemokines expression**	**Cell adhesion molecules**	**Endotoxin contamination**	**References**
APP	Enhance activation of macrophages	NO^↑^	IFN-γ^↑^			NT	(30)
	Murine splenocytes proliferation^↑/↓^		TNF-α^↑^				
PCP	Macrophage activation	iNOS^↑^	TNF-α^↑^, IL-1β^↑^	IL-6^↑^, IL-12^↑^, IL-18^↑^		Neutralized LPS	(28, 55)
	Mouse splenocytes proliferation		IL-2^↑^, IFN-γ^↑^, TNF-α^↑^, IL-4^N^, IL-5^N^	IL-4^↑^, IL-5^↑^	CD44 (T cells)^↑^, CD69 (T cells)^↑^		
ACA	M1 polarization and differentiation	iNOS^↑^, NO^↑^	TNF-α^↑^, IL-1β^↑^, IL-12^↑^	TNF-α^↑^, IL-1β^↑^, IL-6^↑^, IL-12^↑^, IL-10^N^, CCL3^↑^, CCL4^↑^, CCL5^↑^, CCL10^↑^, CCL17^N^, CCL22^N^, CCL24^N^	MHCII^↑^, CD86^↑^, CD80^N^	NT	(26)
PCiP	Macrophage activation	NO^↑^	TNF-α^↑^			NT	(32)
	Murine splenocytes proliferation^↑/↓^		IFN-γ^↑^				
TVC	Enhance macrophage activation	NO^↑^	TNF-α^↑^			NT	(34)
	Enhance the proliferation of splenocytes and hPBLs						
TFP	M1-polarization		TNF-α^↑^, IL-1β^↑^, IL-1ra^↑^, IL-12^↑^	CCL3^↑^, CXCL10^↑^, CCL4^N^, CCL5^N^, CCL17^N^, CCL24^N^	CD86^↑^, MHCII^↑^, CD80^N^	0.14 EU/mg	(29)
PEP 1b	M1-polarization	NO^↑^, iNOS^↑^	TNF-α^↑^, IL-1β^↑^, IL-6^↑^, IL-8^N^			0.570 ± 0.085 EU/mL	(33)
YZP	B cell activation and differentiation		IL-6^↑^, IL-10^↑^		CD1d^↑^, CD25^↑^, CD69^↑^	Less than 0.013 EU/mg	(27)
	Suppress LPS-activated macrophage		TNF-α^↓^, IL-1β^↓^	TNF-α^↓^, IL-1β^↓^, IL-6^↓^, IL-12^↓^, IL-10^↑^			
HEP3	Splenocytes proliferation, and T cells proliferation and differentiation		GM-CSF^↑^, IFN-γ^↑^, IL-4^↑^, IL-12^↑^, IL-17^↑^, TNF-α^↓^, IL-10^↓^, VEGF^↓^		CD3^↑^, CD4^↑^, CD8^↑^, CD28^↑^	NT	(31)
	Suppress LPS-activated macrophage	NO^↓^, iNOS^↓^	TNF-α^↓^, IL-1β^↓^, IL-6^↓^				

↑ *In the superscript means up-regulation*.

↓ *In the superscript means down-regulation*.

N *In the superscript means no significant change*.

↑/↓ *Indicates that APP and PCiP can proliferate splenocytes, but suppress concanavalin A-induced proliferation in vitro (30, 32)*.

*NT means no test mentioned in the reference*.

*(Cells) in which the adhesion molecules were detected*.

#### Induction of LPS-Mimicking Pro-Inflammatory Response

LPS and other endotoxins polarize macrophages toward the M1 phenotype by binding to TLR4 and activating downstream signaling (92). This would lead to the induction of nitric oxide (NO) synthase and secretion of TNF-α and IL-6, which might contribute to increased inflammation, immune stimulation, and tumor suppression (93, 94). FIPs in the left side arrow (see Figure 4) could activate macrophages, or enhance the LPS-induced pro-inflammatory response toward macrophages (APP and TVC). Specifically, these FIPs could induce NO secretion or inducible nitrogen oxide synthase (iNOS) expression; up-regulate production of pro-inflammatory cytokines or chemokines; and enhance synthesis of cell adhesion molecules, phagocytosis, as well as M1 polarization (Table 4). Mechanistically, TLR2/myeloid differentiation primary response (MyD)88 may play a critical role in ACA-induced macrophage activation and polarization (26). The TLR2-triggered NF-κB activation would lead the expressions of express M1 related genes (26). PCP, TFP, and PEP 1b all rely on the TLR4-mediated NF-κB signaling pathway with the involvement of MyD88 to induce expression of cytokines and chemokines in macrophages (28, 29, 33). In these specimens, endotoxins in PCP were neutralized (28); though PEP 1b could induce NO production without endotoxin (33), the content of endotoxin in PEP 1b and TFP (see Table 4) might have contributed to activation of macrophages via the TLR4 signaling pathway or enhanced its activation (74, 75). Meanwhile, PEP1b depended on the MAPK signaling pathway as well. Since TNF receptor-associated factor (TRAF)6 is involved in both TLR2/NF-κB and TLR4/NF-κB signaling pathways (95–98), it may be the connecting factor in these two signaling pathways (see Figure 4).

These FIPs also show effects on splenocytes. APP and PCiP both can activate murine splenocytes, but suppress concanavalin A-induced proliferation of splenocytes *in vitro* (30, 32). The mechanism for this may be competition for the same mediators in signaling pathways (30, 32). Besides, PCiP may decrease 3-(4,5-dimethyldiazol-2-yl)-2,5 diphenyl tetrazolium bromide (MTT) metabolism of concanavalin A-induced splenocytes without the induction of cytotoxicity (32). TVC enhanced the proliferation of splenocytes and hPBLs, while it could not proliferate CD4^+^ and CD8^+^ T cells (34). However, PCP could activate CD8^+^ T cells, induce the secretion of IFN-γ and IL-2 in CD4^+^ T cells, as well as regulate Th1/Th2 response. More specifically, oral-treatment with PCP could increase IgG2a and Th1-related cytokines in splenocytes, and decrease IgG1, IgE, and Th2-related cytokines in an atopic dermatitis mice model. Further, PCP increased T-bet (a transcription factor that stimulates Th1 response) while inhibiting Th2 response (99) and STAT4 (Th1 transcription factor) expression (55). These observations indicate that PCP might be a regulator of Th1/Th2 balance that favors the Th1 response.

#### Anti-inflammatory and Anti-tumor Effects

YZP and HEP3 could reduce activation of LPS-activated macrophages, suppress inflammatory cytokine expression, and show other anti-inflammatory and anti-tumor effects as well (27, 31). YZP could modulate humoral immunity through regulating B-cell activation and suppressing macrophages via B cell in a mixed leukocyte reaction. More specifically, it can enhance IL-10 and IgM secretion, CD25, CD69, and CD1d expression in B cells, and trigger B-reg differentiation, for which the signaling pathway relies on TLR2/NF-κB and TLR4/NF-κB (see Figure 4). For instance, YZP was tested as a colonic inflammatory therapy on an acute colitis murine model, and the effects observed were primarily by B cell regulation (27). Comparably, HEP3 showed stronger potential as a colonic inflammatory treatment, and even in tumor therapy (31). It could suppress LPS-activated macrophages by reducing inflammatory cytokines and downregulating the expression of inducible NO synthase and NK-κB p65 *in vitro*. Further investigation showed that HEP3 could induce proliferation and differentiation of T cells via the gut microbiota, stimulate intestinal APCs in an inflammatory model in mice, and act as anti-tumor inhibitor via an immune mechanism in tumor-grafted mice.

### Hemagglutination Properties of FIPs

Quite a few types of FIPs can agglutinate mouse or rat, rabbit, sheep, or human red blood cells (RBCs) (see Table 5). There are some conflicting data, and some variations in e.g., threshold values in the chart, maybe because of interference of artifacts. In the hemagglutination test, concanavalin A and phytohemagglutinin (both lectins from legume plants) are commonly used as positive control while PBS is always used as a negative control. Since lectin recognition of sugars is rather specific, most agglutination reactions induced by lectins can be inhibited by monosaccharides or oligosaccharides (106). Nevertheless, no inhibition of hemagglutination via several kinds of mono- and disaccharides has been described in FIP-fve (4), LZ-8 (6), APP (30), respectively. FIPs resemble lectins in their hemagglutinating properties but in other features they act clearly different from lectins (12). FIPs may interact with other types of saccharides, or the mechanism behind FIP-induced agglutination is different. Specifically, Fve-type FIPs have a putative carbohydrate binding module (CBM)34-like structure (for details, please see CBM34-Like Structure) linked to glycan chain-binding properties (69).

**Table 5:** The FIPs concentration required [μg/ml] to hemagglutinate different origins of RBCs.

**FIPs**	**Origin of RBCs**				**References**
	**Mouse/(rat)**	**Rabbit**	**Sheep**	**Human**	
LZ-8	NT	NT	6.25	F	(84)
rLZ-8 (*E. coli*)	10	NT	NT	F	(100)
rLZ-8 (*P. pastoris*)	10/1.25	2.5	12.5/20	0.16 (O type)	(12, 100, 101)
rLZ-9 (*P. pastoris*)	1.25	2.5	5	0.156 (O type)	(12)
FIP-fve	NT	NT	F	2/12.5	(4, 71)
rFIP-fve (*E. coli*)	NT	NT	NT	2 (O type)	(102)
rFIP-fve (*P. pastoris*)	F	5	F	2	(12, 103)
OsDp2Fve (rice cell)	NT	NT	NT	F	(104)
FIP-vvo	1.1/0.52 (rat)	0.13	NT	F	(24)
rFIP-vvo (*P. pastoris*)	0.2 (rat)	NT	1.0	F	(105)
rFIP-nha (*P. pastoris*)	0.625	5	0.62	0.16 (O type)	(12)
rFIP-nha (*E. coli*)	NT	1.28	NT	100	(72)
FIP-lrh (*E. coli*)	5	NT	NT	5	(20)
FIP-cru	2	NT	2	F	(53)
rFIP-gap1 (*P. pastoris*)	1	NT	1	1	(10)
rFIP-gap2 (*P. pastoris*)	8	NT	8	8	(10)
rFIP-ppl (*E. coli*)	NT	64	NT	F	(9)
rFIP-tvc (*E. coli*)	1/2 (rat)	NT	NT	NT	(23)
APP	4	NT	NT	NT	(30)
ACA	F	NT	NT	F	(26)
PciP	F	NT	NT	NT	(32)
PCP	F	NT	NT	NT	(28)
TFP	F	NT	NT	NT	(29)
TVC	F	NT	NT	NT	(34)

*F means fail to aggregate the red blood cell*.

*NT means not tested*.

## Structure and Function Relationship

The protein structures of Fve-type FIPs are all modeled based on the crystal structures of FIP-fve (PDB code: 1OSY), LZ-8 (PDB code: 3F3H), and FIP-gmi (PDB code: 3KCW). No other FIP-structures have been described yet. Evaluation of FIPs' bioactivity has been done with many read-out systems. Therefore, no conclusive conclusions can be drawn so far. However, the impact of key residues, CBM34-like structure, oligomeric states, and glycosylation have been described to some extent.

### CBM34-Like Structure

**Figure 5:**
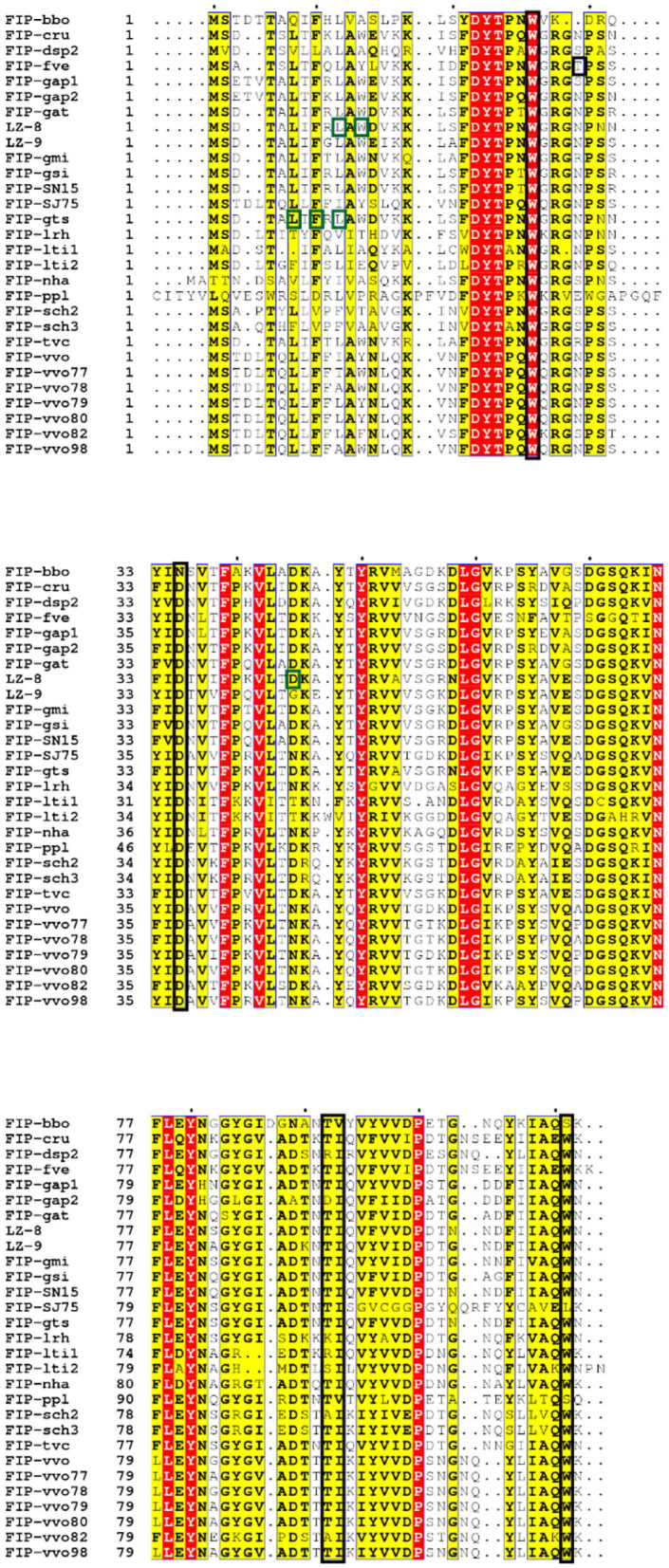
Sequence alignment of Fve-type FIPs (Table 1) via ESPript (http://espript.ibcp.fr/ESPript/cgi-bin/ESPript.cgi) with vital molecules or domains indicated [adapted from (110)]. The black blocked amino acids may be essential for hemagglutination and induction of IFN-γ in hPBMCs, which has been investigated on FIP-fve (W24, T28, D34, T90, I91, and W111) (69) and confirmed on FIP-lrh (W25, D35, I92, and W110) (20); the green blocked units are imported for IL-2 expression induced by LZ-8 (L10, W12, and D45) (110) or IL-2 and IFN-γ secretion induced by FIP-gts (L5, F7, and L9) (17).

CBM structures always exist in glycoside hydrolases to promote substrate association (107). The CBM family has 86 members listed in CAZy (http://www.cazy.org/) (108). FIP-fve mimics CBM34, a β-sandwich folding family that shows granule starch-binding functionality (109). W24, T28, D34, T90, I91, and W111 of FIP-fve (see Figures 2A, 5) are the key residues for the CBM34-like structure formation (69). This structure may contribute to hemagglutination and induction of IFN-γ secretion in hPBMCs (69). Hemagglutination activity seems to be specifically related to W24, D34, I91, and W111, while the immunomodulatory activity is more associated with W24, D34, T90, and W111 that are essential for ligand-like glycoproteins binding interaction on the surface of hPBMCs (69). Liu et al. also tested the inhibition effects of 15 kinds of saccharides in hPBMCs: N-acetylneuraminic acid, maltotriose, cyclodextrin, and dextrin could inhibit IFN-γ secretion significantly (69). This suggests that the CBM34-like structure might have a preference in sugar binding. Interestingly, 6 kinds of mono- and disaccharides could not inhibit hemagglutination by FIP-fve (4). Amid these saccharides, N-acetylgalactosamine could weakly block FIP-fve-induced IFN-γ production of hPBMCs at 30 mM, while there is no impact on hemagglutination even at 0.1 M (4, 69).

In the amino acid composition of the predicted CBM34-like structure, T28 is only present in FIP-fve, while there are small position shift or residues variation in other Fve-type FIPs (Figure 5), which may impact their hemagglutination (Table 5) and IFN-γ inducing properties (Table 3). Prediction of the CBM34-like structure has been confirmed in FIP-lrh, consisting of W25, D35, I92, W110, as well as two residues variation, N29 and K90 (see Figure 5). Docking predictions of FIP-lrh and FIP-fve on several glycans (commonly found on cellular surfaces) showed their binding energy, although the binding energy of FIP-lrh is lower than FIP-fve (20). Evidentially, the threshold of FIP-fve on human erythrocyte agglutination was 2 μg/mL, while the threshold of FIP-lrh was up to 5 μg/mL (20). Although not only the structure contributes to erythrocytes agglutination, the glycan type on the erythrocyte cell surface can also influence the agglutination. This may explain why the same FIPs behave differently on erythrocytes from different species, and that there is variation in thresholds for various types of FIPs on the same type of erythrocytes (see Table 5). Meanwhile, IFN-γ secretion in hPBMCs induced by FIP-fve is related to the interaction with glycans on the cell surface and the supposed CBM34-like structure (69). It is tempting to investigate cell surface glycosylation further and to see whether bioactivities of FIPs can be affected by such modifications.

### Oligomeric States

Some articles hint that the dimerization state of Fve-type FIPs plays a vital role in their activity. For example, the monomer of FIP-gts did not induce cytokine production on hPBLs (17, 111). L5, F7, L9, and the N-terminal α-helix of Fip-gts, which affects its dimerization, are essential in inducing IL-2 and IFN-γ secretion (17). L10, W12, and D45, which are located in the interface of dimerized LZ-8, are pivotal in triggering IL-2 secretion on hPBLs (110). Furthermore, the crystal structures of FIP-fve (43, 112) and LZ-8 (113) also show that the N-terminal α-helix is highly conserved in Fve-type FIPs, and the conserved N-terminal β-sheet may sustain the dimerization state via domain swapping (46).

### Glycosylation Modification

The effects of glycosylation of FIPs manifest in the induction of cytokine production/secretion, cell adhesion molecules expression, and lymphocyte proliferation. For instance, the level of IL-2 induction resulting from the non-glycosylated rFIP-gts was lower than for the glycosylated form (8, 114), indicating that the post-translational processing of rFIP-gts might play an essential role in enhancing and maintaining the required immunomodulatory activity on T lymphocytes. Besides, there were more noticeable cellular lymphocytes aggregates of murine splenocytes when treated with glycosylated rFIP-gts than with the non-glycosylated form (8). As reported in literature, hPBL cellular aggregation coincides with the upregulation of ICAM-1 expression and T cell proliferation (115–117). Therefore, the glycosylated rFIP-gts might be more potent in the induction of ICAM-1 expression and T cell proliferation.

Comparably, only native PCP (glycosylated fraction) was able to increase the cell surface expression of TLR4 in peritoneal macrophages from wild type mice; deglycosylated PCP, or LPS cannot (28). This evidence indicated that PCP-induced macrophage activation was directly correlated with the polysaccharide moiety of PCP. In contrast, in Sheu's research, both the native ACA (glycoprotein) and rACA (expressed by *E. coli*) showed dose-dependent induction of TNF-α in macrophages, and the level of TNF-α induction was higher in response to rACA than to native ACA (26). Similar activation differences were observed for IL-1β and NO by the presence of ACA (26). The most apparent difference between PCP and ACA in activating macrophages is in the involved receptors on the cell surface. PCP activates macrophages via the TLR4/MyD88 signaling pathway (28), while ACA activates macrophages via the TLR2/MyD88 signaling pathway and induces M1 polarization and differentiation (26). There is no evidence showing that FIPs are taken up by a macrophage, and the TLR2/MyD88 as well as TLR4/MyD88 signaling pathway will not induce endocytosis (97, 98). The different sensitivity of TLR4 and TLR2 for activation of the signaling pathway, when being exposed to glycosylated or other types of FIPs, may play a role. Hence, more research is needed to elucidate the further mechanisms of FIPs and the role of glycosylation. Another kind of explanation may be LPS contamination, as rACA was expressed in *E. coli*, and no LPS investigation or removal method is mentioned in their study (26).

## Prospects

Fungi contain many bioactive molecules, including polysaccharides, lectins, and FIPs. Until now, more than 38 types of FIPs have been described, which can be divided into 5 subgroups. However, the list of Fve-type FIPs and Cerato-type FIPs could be expanded in the future. For instance, FIP-gja from *Ganoderma japonicum* (Genbank: AAX9824) could be a Fve-type FIP, ACA2 from *Antrodia camphorate* (GenBank: ABE01080) and TVCs (GenBank: EIW60955.1, EIW60914.1, EIW60949.1) from *T. versicolor* would be expected to belong to Cerato-type FIPs.

Fve-type FIPs are highly interesting because of their anti-allergic, anti-inflammatory, and anti-cancer bioactivity, especially as they have not shown side-effects in allergy treatment or as an adjuvant to attack drug-resistant tumor cells based on current findings. Co-treatment of medicine and Fve-type FIPs is important to investigate as well, since Fve-type FIPs have shown intense immunomodulatory activity and promising options in drug-resistant tumor cells. Research has shown that some Fve-type FIPs can retain their bioactivity upon oral administration. That means they are rather digestion-resistant, or that some vital domain, responsible for their bioactivity, reaches the intestinal tract intact. In some articles, the thermal stability and digestion resistance of FIPs have been described (111, 118–120). Additional research will deliver details about essential domains as well as their utilization.

Regarding the other four subgroups, their bioactivity is obviously worth further study. Those FIPs which can induce/enhance an LPS-mimetic response could be used as an adjuvant to enhance the immune response in the host. FIPs that can suppress an LPS-induced response could be used in anti-inflammatory therapy. Two types of FIPs show benefits for reducing colonic inflammation, and even toward cancer via the gut microbiota. It will be meaningful and useful to deeper investigate their mechanisms and applications.

Up to now, only three Fve-type FIP structures have been solved. Several articles show protein structure and function relationships and use the amino acid sequence to predict their function or activity (121–123). This suggests that more structural information on Fve-type FIPs, as well as on the other subgroups of FIPs, will increase comprehension of their bioactivity. Although a precise mechanism is still unknown, there are relevant illustrations of some structural-related bioactive functions. For instance, FIP-gts (124), FIP-nha (125), and LZ-8 (126) can be transported into tumor cells and exert toxicity, and LZ-8 could even enter the nucleus of NB4 cells (127). The way they distinguish tumor cells and normal cells, the vital structure which enters the cell and behaves toxically, and the mechanism of their anti-tumor activity need further investigation. Regarding the glycosylation modification, multiple results indicate that it may influence FIPs' bioactivity, which has been describing in “4.3 Glycosylation modification.” Meanwhile, there are more FIPs shown in Table 2 with potential glycosylation sites, which could be further investigated to shed further light on the influence of glycosylation on bioactivity of FIPs systematically.

## Author Contributions

YL and SB-N wrote the first draft of the paper. All authors read and contributed to the final version of the paper.

## Conflict of Interest

The authors declare that the research was conducted in the absence of any commercial or financial relationships that could be construed as a potential conflict of interest.

## Abbreviations

APCs: antigen-presenting cells
CD: cluster of differentiation
D: aspartic acid
DCs: Dendritic cells
ERK: extracellular signal-regulated kinase
F: phenylalanine
FIP: fungal immunomodulatory protein
GM-CSF: Granulocyte-macrophage colony-stimulating factor
HLA: human leukocyte antigen
hPBL: human peripheral blood lymphocyte
I: isoleucine
IFN: interferon
ICAM: intercellular cell adhesion molecule
Ig: immunoglobulin
IL: interleukin
IL-2R: interleukin-2 receptor
L: leucine
LPS: lipopolysaccharide
LT: lymphotoxin
MAPK: mitogen-activated protein kinase
MHC II: major histocompatibility complex
Mr: relative molecular mass
MyD88: myeloid differentiation primary response 88
N: asparagine
NF-kB: nuclear factor-kB
NO: nitric oxide
PBL: peripheral blood lymphocyte
PBMC: peripheral blood mononuclear cell
PKC: protein kinase C
PLC: phospholipase C
PTK: protein tyrosine kinase
ROS: reactive oxygen species
RSV: respiratory syncytial virus
S: serine
T: threonine
Th: T helper
TLR: Toll-like receptor
TNF: tumor necrosis factor
TRAF: TNF receptor-associated factor
VEGF: vascular endothelial growth factor
W: tryptophan.
