# Supplementary material for: Current Understanding of the Structure and Function of Fungal Immunomodulatory Proteins
Source: Front Nutr. 2020 Aug 18;7:132. doi: 10.3389/fnut.2020.00132 (PMC7461872; doi:10.3389/fnut.2020.00132)
Supplement: Supplementary file 1 [file Table_1.DOCX]

Supplementary Material

**Supplementary Table 1.** Structure and characteristics of FIPs

| name | Amino acid residues | Secondary structure composition | Oligomerization State | Molecular Weight (kDa) | Isoelectric point (pI) | Reference |
| --- | --- | --- | --- | --- | --- | --- |
| **Fve-type FIPs** |  |  |  |  |  |  |
| LZ-8 | 110 | 2H+7E | homodimer | 13.1 | 4.4 | (Tanaka et al., 1989; Kino et al., 1990; An et al., 2010) |
| LZ-9 | 111 | 2H+7-9E* | homodimer | 12.4 | 4.33 | (Bastiaan-Net et al., 2013) |
| FIP-fve | 114 | 1H+8E | homodimer | 12.7 | 6.17^#^ | (Ko et al., 1995; Hsieh et al., 2003; Paaventhan et al., 2003) |
| FIP-vvo | 112 | 2H+8E | homodimer | 12.7 | 7.86^#^ | (Hsu et al., 1997; Wang et al., 2016) |
| FIP-vvos | 113 | 1-2H+7-8E | homodimer | ~13 | 6.06~8.83 | (Wang et al., 2016) |
| FIP-gts | 110 | 2H+7E | homodimer | 12.5* | 4.84* | (Lin et al., 1997) |
| FIP-cru | 113 | 2H+7-9E* | homodimer | 12.7 | 6.81 | (Lin et al., 2016) |
| FIP-dsp2 | 111 | 2H+7E | homodimer | 12.5 | 6.05 | (Li et al., 2017a) |
| FIP-tvc | 111 | 2H+7-9E* | homodimer | 12.4 | 4.81 | (Li et al., 2012) |
| FIP-gap1 | 113 | 2-3H+6E | homodimer | 12.7 | 4.93 | (Zhou et al., 2018) |
| FIP-gap2 | 113 | 2-3H+7-9E | homodimer | 12.5 | 4.86 | (Zhou et al., 2018) |
| FIP-gat | 111 | 1-2H+8E* | homodimer | 12.5 | 4.80 | (Xu et al., 2016) |
| FIP-gmi | 111 | 2H+7E | tetramer | 15.9 | 4.58^#^ | (Wu et al., 2007) |
| FIP-lrh | 112 | 2H+7E | homodimer | 12.6 | 6.54^#^ | (Pushparajah et al., 2016) |
| FIP-lti1 | 113 | 2H+7E | homodimer | 12.61 | 8.66 | (Gao et al., 2019) |
| FIP-lti2 | 115 | 2H+7E | homodimer | 12.80 | 5.49 | (Gao et al., 2019) |
| FIP-nha | 114 | 2H+8E* | homodimer | 12.8 | 9.25 | (Bastiaan-Net et al., 2013) |
| FIP-ppl | 125 | 2H+7E | homodimer | 14.5 | 5.50^#^ | (Li et al., 2015) |
| FIP-sch2 | 112 | 2H+7E | homodimer | 12.6 | 6.05 | (Li et al., 2017b) |
| FIP-gsi | 111 | 1H+8E | homodimer | 12.4 | 4.81 | (Zhou et al., 2009) |
| FIP-SJ75 | 115 | 2H+7E | homodimer | 12.9 | 7.68 | (Shao et al., 2019) |
| FIP SN15 | 111 | 2H+7E | homodimer | 12.6* | 4.66* | (Cong et al., 2014) |
| **Cerato-type FIPs** |  |  |  |  |  |  |
| ACA | 136(18aa SP) | 6H+7E* | monomer | 28.7 | 3.39* | (Sheu et al., 2009) |
| YZP | 139(19aa SP) | 3-4H+5E* | monomer | 12.1 | 4.19* | (Kuan et al., 2013) |
| **PCP-like FIPs** |  |  |  |  |  |  |
| PCP | 193 | 2H+5E* | heterodimer | 35.6 | 5.2 | (Chang et al., 2009; Lu et al., 2014) |
| **TFP-like FIPs** |  |  |  |  |  |  |
| TFP | 112 | 2H+6E* | homodimer | 24 | 3.5 | (Hung et al., 2014) |
| **Unclassified FIPs** |  |  |  |  |  |  |
| APP | - | - | - | 13.4 | 5.1 | (Sheu, 2004) |
| PCiP | - | - | - | 15 | 5.2 | (Sheu, 2010) |
| TVC | - | - | dimer | 30 | 4.0 | (Li et al., 2011) |
| HEP3 | - | - | - | 52 | - | (Diling et al., 2017) |
| PEP 1b | - | - | - | 21.9 | - | (Hu et al., 2018) |

*means based on prediction modelling. pI is predicted by <https://web.expasy.org/compute_pi/>; secondary structure is predicted by <http://scratch.proteomics.ics.uci.edu/index.html>, H indicates helix; E indicates β-sheet; pI and Molecular weight is predicted by <https://web.expasy.org/protparam/>.

**Reference**

An, M., Gao, G.F., Qi, J., Li, F., and Liu, X. (2010). Expression and crystallographic studies of a fungal immunomodulatory protein LZ-8 from a medicinal fungus *Ganoderma lucidum*. *Sheng Wu Gong Cheng Xue Bao* 26(11)**,** 1563-1568.

Bastiaan-Net, S., Chanput, W., Hertz, A., Zwittink, R.D., Mes, J.J., and Wichers, H.J. (2013). Biochemical and functional characterization of recombinant fungal immunomodulatory proteins (rFIPs). *Int Immunopharmacol* 15(1)**,** 167-175. doi: 10.1016/j.intimp.2012.11.003.

Chang, H.H., Yeh, C.H., and Sheu, F. (2009). A novel immunomodulatory protein from *Poria cocos* induces Toll-like receptor 4-dependent activation within mouse peritoneal macrophages. *J Agric Food Chem* 57(14)**,** 6129-6139. doi: 10.1021/jf9011399.

Cong, W.R., Xu, H., Liu, Y., Li, Q.Z., Li, W., and Zhou, X.W. (2014). Production and functional characterization of a novel fungal immunomodulatory protein FIP-SN15 shuffled from two genes of *Ganoderma* species. *Appl Microbiol Biotechnol* 98(13)**,** 5967-5975. doi: 10.1007/s00253-014-5539-4.

Diling, C., Chaoqun, Z., Jian, Y., Jian, L., Jiyan, S., Yizhen, X., et al. (2017). Immunomodulatory activities of a fungal protein extracted from *Hericium erinaceus* through regulating the gut microbiota. *Front Immunol* 8**,** 666. doi: 10.3389/fimmu.2017.00666.

Gao, Y., Wang, Y., Wang, Y., Wu, Y., Chen, H., Yang, R., et al. (2019). Protective function of novel fungal immunomodulatory proteins Fip-lti1 and Fip-lti2 from *Lentinus tigrinus* in concanavalin A-induced liver oxidative injury. *Oxid Med Cell Longev* 2019**,** 3139689. doi: 10.1155/2019/3139689.

Hsieh, K.Y., Hsu, C.I., Lin, J.Y., Tsai, C.C., and Lin, R.H. (2003). Oral administration of an edible-mushroom-derived protein inhibits the development of food-allergic reactions in mice. *Clin Exp Allergy* 33(11)**,** 1595-1602.

Hsu, H.C., Hsu, C.I., Lin, R.H., Kao, C.L., and Lin, J.Y. (1997). Fip-vvo, a new fungal immunomodulatory protein isolated from *Volvariella volvacea*. *Biochem J* 323 ( Pt 2)**,** 557-565.

Hu, Q., Du, H., Ma, G., Pei, F., Ma, N., Yuan, B., et al. (2018). Purification, identification and functional characterization of an immunomodulatory protein from *Pleurotus eryngii*. *Food Funct* 9(7)**,** 3764-3775. doi: 10.1039/c8fo00604k.

Hung, C.L., Chang, A.J., Kuo, X.K., and Sheu, F. (2014). Molecular cloning and function characterization of a new macrophage-activating protein from *Tremella fuciformis*. *J Agric Food Chem* 62(7)**,** 1526-1535. doi: 10.1021/jf403835c.

Kino, K., Mizumoto, K., Sone, T., Yamaji, T., Watanabe, J., Yamashita, A., et al. (1990). An immunomodulating protein, Ling Zhi-8 (LZ-8) prevents insulitis in non-obese diabetic mice. *Diabetologia* 33(12)**,** 713-718.

Ko, J.L., Hsu, C.I., Lin, R.H., Kao, C.L., and Lin, J.Y. (1995). A new fungal immunomodulatory protein, FIP-fve isolated from the edible mushroom, *Flammulina velutipes* and its complete amino acid sequence. *Eur J Biochem* 228(2)**,** 244-249.

Kuan, Y.C., Wu, Y.J., Hung, C.L., and Sheu, F. (2013). *Trametes versicolor* protein YZP activates regulatory B lymphocytes - gene identification through de novo assembly and function analysis in a murine acute colitis model. *PLoS One* 8(9)**,** e72422. doi: 10.1371/journal.pone.0072422.

Li, F., Wen, H., Liu, X., Zhou, F., and Chen, G. (2012). Gene cloning and recombinant expression of a novel fungal immunomodulatory protein from *Trametes versicolor*. *Protein Expr Purif* 82(2)**,** 339-344. doi: 10.1016/j.pep.2012.01.015.

Li, F., Wen, H., Zhang, Y., Aa, M., and Liu, X. (2011). Purification and characterization of a novel immunomodulatory protein from the medicinal mushroom *Trametes versicolor*. *Sci China Life Sci* 54(4)**,** 379-385. doi: 10.1007/s11427-011-4153-2.

Li, S., Jiang, Z., Sun, L., Liu, X., Huang, Y., Wang, F., et al. (2017a). Characterization of a new fungal immunomodulatory protein, FIP-dsq2 from *Dichomitus squalens*. *J Biotechnol* 246**,** 45-51. doi: 10.1016/j.jbiotec.2017.02.006.

Li, S., Jiang, Z., Xu, W., Xie, Y., Zhao, L., Tang, X., et al. (2017b). FIP-sch2, a new fungal immunomodulatory protein from *Stachybotrys chlorohalonata*, suppresses proliferation and migration in lung cancer cells. *Appl Microbiol Biotechnol* 101(8)**,** 3227-3235. doi: 10.1007/s00253-016-8030-6.

Li, S.Y., Shi, L.J., Ding, Y., Nie, Y., and Tang, X.M. (2015). Identification and functional characterization of a novel fungal immunomodulatory protein from *Postia placenta*. *Food Chem Toxicol* 78**,** 64-70. doi: 10.1016/j.fct.2015.01.013.

Lin, J.W., Guan, S.Y., Duan, Z.W., Shen, Y.H., Fan, W.L., Chen, L.J., et al. (2016). Gene cloning of a novel fungal immunomodulatory protein from *Chroogomphis rutilus* and its expression in *Pichia pastoris*. *Journal of Chemical Technology & Biotechnology* 91(11)**,** 2761-2768.

Lin, W.H., Hung, C.H., Hsu, C.I., and Lin, J.Y. (1997). Dimerization of the N-terminal amphipathic alpha-helix domain of the fungal immunomodulatory protein from *Ganoderma tsugae* (Fip-gts) defined by a yeast two-hybrid system and site-directed mutagenesis. *J Biol Chem* 272(32)**,** 20044-20048.

Lu, Y.T., Kuan, Y.C., Chang, H.H., and Sheu, F. (2014). Molecular cloning of a *Poria cocos* protein that activates Th1 immune response and allays Th2 cytokine and IgE production in a murine atopic dermatitis model. *J Agric Food Chem* 62(13)**,** 2861-2871. doi: 10.1021/jf405507e.

Paaventhan, P., Joseph, J.S., Seow, S.V., Vaday, S., Robinson, H., Chua, K.Y., et al. (2003). A 1.7A structure of Fve, a member of the new fungal immunomodulatory protein family. *J Mol Biol* 332(2)**,** 461-470.

Pushparajah, V., Fatima, A., Chong, C.H., Gambule, T.Z., Chan, C.J., Ng, S.T., et al. (2016). Characterisation of a new fungal immunomodulatory protein from tiger milk mushroom, *Lignosus rhinocerotis*. *Sci Rep* 6**,** 30010. doi: 10.1038/srep30010.

Shao, K.-D., Mao, P.-W., Li, Q.-Z., Li, L.-D.-J., Wang, Y.-l., and Zhou, X.-W. (2019). Characterization of a novel fungal immunomodulatory protein, FIP-SJ75 shuffled from *Ganoderma lucidum*, *Flammulina velutipes* and *Volvariella volvacea*. *Food and Agr Immunol* 30(1)**,** 1253-1270. doi: 10.1080/09540105.2019.1686467.

Sheu, F., Chien, P.J., Hsieh, K.Y., Chin, K.L., Huang, W.T., Tsao, C.Y., et al. (2009). Purification, cloning, and functional characterization of a novel immunomodulatory protein from *Antrodia camphorata* (bitter mushroom) that exhibits TLR2-dependent NF-kappaB activation and M1 polarization within murine macrophages. *J Agric Food Chem* 57(10)**,** 4130-4141. doi: 10.1021/jf900469a.

Sheu, F., Chien, P. J. , Chien, A. L. , Chen, Y. F. , & Chin, K. L. (2004). Isolation and characterization of an immunomodulatory protein (app) from the jew's ear mushroom auricularia polytricha. *Food Chem* (87(4))**,** 593-600. doi: 10.1016/j.foodchem.2004.01.015.

Sheu, F., Chien, P. J. , Wang, H. K. , Chang, H. H. , & Shyu, Y. T. (2010). New protein PCiP from edible golden oyster mushroom pleurotus citrinopileatus activating murine macrophages and splenocytes. *J Sci Food Agric* (87(8))**,** 1550-1558. doi: 10.1002/jsfa.288710.1002/jsfa.

Tanaka, S., Ko, K., Kino, K., Tsuchiya, K., Yamashita, A., Murasugi, A., et al. (1989). Complete amino acid sequence of an immunomodulatory protein, ling zhi-8 (LZ-8). An immunomodulator from a fungus, *Ganoderma lucidium*, having similarity to immunoglobulin variable regions. *J Biol Chem* 264(28)**,** 16372-16377.

Wang, Y., Wa Ng, Y., Gao, Y., Li, Y., Wan, J.N., Yang, R.H., et al. (2016). Discovery and characterization of the highly active fungal immunomodulatory protein Fip-vvo82. *J Chem Inf Model* 56(10)**,** 2103-2114. doi: 10.1021/acs.jcim.6b00087.

Wu, M., Hsu, M., Huang, C.S., Fu, H., Huang, C.T., and Yang, C.S. (2007). A 2.0 Å structure of GMI, a member of the fungal immunomodulatory protein family from *Ganoderma microsporum*. *Protein crystallogr* 2**,** 132.

Xu, H., Kong, Y.Y., Chen, X., Guo, M.Y., Bai, X.H., Lu, Y.J., et al. (2016). Recombinant FIP-gat, a fungal immunomodulatory protein from *Ganoderma atrum*, induces growth inhibition and cell death in breast cancer cells. *J Agric Food Chem* 64(13)**,** 2690-2698. doi: 10.1021/acs.jafc.6b00539.

Zhou, S., Guan, S., Duan, Z., Han, X., Zhang, X., Fan, W., et al. (2018). Molecular cloning, codon-optimized gene expression, and bioactivity assessment of two novel fungal immunomodulatory proteins from *Ganoderma applanatum* in *Pichia*. *Appl Microbiol Biotechnol* 102(13)**,** 5483-5494. doi: 10.1007/s00253-018-9022-5.

Zhou, X., Xie, M., Hong, F., and Li, Q.Z. (2009). Genomic cloning and characterization of a FIP-gsi gene encoding a fungal immunomodulatory protein from *Ganoderma sinense* (Aphyllophoromycetideae). *Int J Med Mushrooms* 11((1)).
